# Competing Values in Global Health: Is Inclusive Governance Valued Higher Than the Right to Health? A Response to the Recent Commentaries

**DOI:** 10.34172/ijhpm.2022.7209

**Published:** 2022-03-13

**Authors:** Mao Suzuki, Roy Small, Douglas Webb

**Affiliations:** ^1^Yale-NUS College, Singapore, Singapore.; ^2^HIV, Health and Development Group, Bureau for Policy and Programme Support, United Nations Development Programme, New York City, NY, USA.

## Introduction

 We thank those who contributed commentaries on our paper “Competing Frames in Global Health Governance: An Analysis of Stakeholder Influence on the Political Declaration on Non-communicable Diseases.” We read them with great interest and appreciate that our study was described as “rare and elaborate” and a “wakeup up call” for additional empirical analysis to understand the various ways that health-harming industries interfere in non-communicable disease (NCD) policy-making, including at country level. This response focuses on two major themes that we identify across the commentaries: interrogation of a broader normative trend in global health governance that values multi-stakeholder processes, potentially at the expense of health and development outcomes, and secondly specific proposals to address the interference of commercial actors in NCD governance.

## A Broader Normative Trend Prioritizing Inclusive Global Health Governance, but at What Cost?

 Our paper showed that competing *frames* across stakeholder groups, along with how these were arbitrated, likely led to a watered-down Political Declaration of the Third High-level Meeting on the Prevention and Control of NCDs. The commentaries suggest that a higher order competition is at play and needs urgent reckoning - that of competing *values* in global health. Carriedo and colleagues^1^ describe the normative trend toward leveraging public-private partnerships (PPPs) for sustainable development, as promulgated through the 2030 Agenda for Sustainable Development. They caution that “While public consultations by the United Nations (UN) are based on the principles of inclusiveness, plurality, and democracy, they have legitimized the participation of powerful commercial actors whose health-harming interests may shape outcome documents.” Despite the prevailing trend toward PPPs and multi-stakeholder processes, Rinaldi^2^ stresses that “there is no clear evidence in favour of the effectiveness of PPPs in public health promotion to date” and, in fact, evidence is growing that unhealthy commodity industries are adopting framing and strategies that are interfering with the implementation of evidence-based public health measures. Carriedo and colleagues^1^ question the underlying assumption that the interests of commercial actors and the public can be aligned. They argue that ambiguity is a defining and strategic feature of multi-stakeholder discourse and that whole-of-society governance obscures the varied interests, motivations, roles and responsibilities of state, civil society and private sector actors, serving to downplay the role and regulation of conflicts of interest (COI).

 Considering that governance responses to NCDs continue to be inadequate worldwide, a shortcoming that has also amplified the severity of the coronavirus disease 2019 (COVID-19) pandemic,^3^ the global community must reflect on the reconcilability of values underpinning global health governance and, in the case of conflicts, determine which values should take precedent. In particular, there is a need to understand how the trend of inclusiveness is serving or undermining the right to health. In assessing and arbitrating potential trade-offs, we encourage the global community to consider the nature of inclusion currently being achieved, particularly in the case of NCD response governance. Ralston^4^ points out that ‘inclusiveness’ is proving to be a misnomer because power imbalances in the consultation processes are skewing outcomes to the benefit of the powerful, and that this is de facto excluding voices of civil society and low- and middle-income countries (LMICs) while perpetuating colonialism in global health. Stronger and more coordinated patient and civil society voices and demands, as advocated by Ralston,^4^ Rinaldi^2^ and Buse et al,^5^ are key to challenging power asymmetries in policy debates. Yet, coalition strengthening is not enough where mere opposition of positions, irrespective of the evidence base or number of advocates, results in weaker commitments, as our paper showed. The rules of the game require revision, and the global community has already shown a willingness to limit inclusion where human rights are jeopardized, for example in preventing the engagement of tobacco and certain weapons industries in various fora.^6,7^

## Strategies to Address Commercial Interference in NCD Governance

 Our paper recommended reconsideration of inclusion/exclusion criteria in consultation processes for global policy-making and governance on NCDs. The commentaries offer promising and practical proposals to that end. To inform decisions around inclusion and exclusion of stakeholders, Buse and colleagues^5^ recommend the development of an index to capture impacts on health and other negative externalities of individual corporate actors based on their products and operations. This process would be facilitated by an independent expert advisory body on public health, corporations and COI, which would also make public, evidence-based recommendations regarding language and proposals made by stakeholders during consultation and negotiation processes. Carriedo and colleagues^1^ also propose an independent committee to scrutinize participation in consultative processes while outlining critical questions for assessing the benefits and appropriateness of multi-stakeholder and PPPs. We strongly endorse such moves to more firmly anchor policy-making to independent, trustworthy and expert consideration of public health impact. We would welcome WHO’s leadership in supporting such directions, as suggested by Buse et al,^5^ and encourage additional coherent support across the multilateral system, for example leveraging the ustainable Development Goal 3 Global Action Plan for Health and Well-being for All.^8^ This would enable enhanced understanding and cross-application of effective strategies in governance for health and development, for example in gender, environment, and pandemic preparedness and response. With respect to the index proposed by Buse et al,^5^ we recommend that any index considers not just the status quo impacts of corporate actors but also directions of corporate behaviour change. Good faith and effective efforts of certain industries to do better should be recognized and rewarded, in line with the principles of the UN Global Compact.^9^

 Berner-Rodoreda and Jahn^10^ argue that UN multi-stakeholder hearings, as a democratic process, should remain open to all stakeholder groups. They point out that even if certain industries are excluded from interactive hearings, they can still exert influence on policy-making through national delegations and intergovernmental negotiations, which are less transparent. Berner-Rodoreda and Jahn are right about this phenomenon, which underscores the importance of their suggestions to establish eligibility criteria for national delegations and to track legislative footprints at country, regional, and UN levels to increase transparency. We believe it also underscores the need for the multilateral system to scale up national and subnational support for effective, multisectoral NCD governance which protects against undue commercial influence in policy-making. Berner-Rodoreda and Jahn’s points in favor of preserving the openness of UN multi-stakeholder processes for NCDs are compelling. However, no matter how the global community ultimately decides on inclusion/exclusion of stakeholders, we reiterate that the status quo – promotion of PPPs together with inadequate COI management of health-harming industries (eg, alcohol, sugar-sweetened beverage and certain food industries) – is the worst possible combination from the standpoint of health and health equity. Zenone and Hawkins^11^ drive this point home in suggesting it is time to move beyond tobacco exceptionalism in extending COI management frameworks and approaches for tobacco to other industries. Given the negative externalities of a wide range of health-harming industries, we agree.

 Finally, we share the view across commentaries, and particularly emphasized by Ralston,^4^ that the clustering of all private sector actors under the umbrella term ‘industry’ is unhelpful and could block potentially positive and innovative solutions for addressing NCDs. In our opinion, however, such private sector solutions have been too slow and too few, often teased in global discourse to stave off restrictions on participation. Any entity or body charged with reassessing inclusion/exclusion criteria must recognize that commercial entities differ across sectors, sizes, localities and interests, and that the private sector has enormous potential to turn the tide on NCDs. Overly exclusive governance processes can have their own fatal flaws, not least lack of ownership and legitimacy. Striking the right balance in stakeholder participation is essential for health and health equity, and the global community must not overcorrect in addressing the deficiencies of inclusive multi-stakeholder processes.

## Conclusion

 In this response, we discuss the normative trend that values multi-stakeholder processes in global health governance at the potential expense of the right to health, and specific ways to reduce commercial interference in NCD policy-making. In concluding we echo the combined calls from Zenone and Hawkins^11^ and Ralston^4^ to decolonize global health by further understanding the power of health-harming industries, addressing industry interference, confronting the failure of high-income countries to learn from LMICs, and ensuring more direct financing flows between health donors and LMIC experts. Our paper showed that high-income countries and the private sector were aligned in generally opposing stricter regulations on commercial factors. Other analyses show that corporate influence or permeation also play a major role at country level in blocking implementation of policies to tackle commercial determinants of NCDs.^12,13^ LMICs continue to be deprived of adequate development assistance for country-led efforts to combat NCDs, with funding for NCDs representing just 1.62% of development assistance for health in 2020.^3^ This must change, and LMICs must no longer be denied the opportunities that stronger, more ambitious and more progressive political declarations can provide.^5^

## Ethical issues

 Not applicable.

## Competing interests

 Authors declare that they have no competing interests.

## Authors’ contributions

 MS structured outlines and wrote a draft. RS conceptualized the response and revised the paper to strengthen points. DW provided comments on drafts. All authors read and approved the final manuscript.
